# Divergent and subnucleus-specific gene expression responses to chronic stress hormone exposure in the amygdala

**DOI:** 10.3389/fnmol.2025.1659846

**Published:** 2025-09-12

**Authors:** Shuhei Ueda, Manami Kakita, Masahito Hosokawa, Koji Arikawa, Kiyofumi Takahashi, Ryusuke Shiota, Masaki Kakeyama, Hiroko Matsunaga, Haruko Takeyama, Sayaka Takemoto-Kimura

**Affiliations:** ^1^Department of Neuroscience, Research Institute of Environmental Medicine, Nagoya University, Nagoya, Japan; ^2^Molecular/Cellular Neuroscience, Nagoya University Graduate School of Medicine, Nagoya University, Nagoya, Japan; ^3^Laboratory for Systems Neurosciences and Preventive Medicine, Faculty of Human Sciences, Waseda University, Tokorozawa, Japan; ^4^Research Institute for Environmental Medical Sciences, Waseda University, Tokorozawa, Japan; ^5^Graduate School of Advanced Science and Engineering, Waseda University, Tokyo, Japan; ^6^Research Organization for Nano and Life Innovation, Waseda University, Tokyo, Japan; ^7^Institute for Advanced Research of Biosystem Dynamics, Waseda Research Institute for Science and Engineering, Waseda University, Tokyo, Japan

**Keywords:** stress, corticosterone, HPA axis, depression, amygdala, central extended amygdala, RNA-seq

## Abstract

Major depressive disorder (MDD) is one of the most prevalent mental disorders, posing a significant socioeconomic burden worldwide. Its development involves both genetic and environmental factors, among which chronic stress is considered a major contributor. The amygdala, a key brain region for emotional regulation, is critically implicated in MDD pathophysiology. Given its complex subnuclear architecture, it is essential to characterize stress-induced molecular changes at the level of individual subnuclei. To investigate subnucleus-specific molecular adaptations to chronic stress, we performed RNA sequencing on fluorescence-guided micropunch samples from five amygdala-related subnuclei in mice exposed to chronic corticosterone (CORT): the basolateral amygdala (BLA), the lateral and medial central amygdala (CeL, CeM), and the oval and fusiform bed nuclei of the stria terminalis (BNSTov, BNSTfu). Comparative transcriptomic analysis revealed highly divergent and subnucleus-resolved gene expression responses to chronic CORT exposure. Each subregion exhibited unique profiles of differentially expressed genes, implicating alterations in excitatory–inhibitory synaptic balance, glial functions involving oligodendrocytes or astrocytes, and neuropeptide signaling. Our results uncover the molecular heterogeneity of subnucleus-specific responses within the amygdala. These findings highlight the importance of anatomically resolved analyses in elucidating the biological basis of stress-related mental disorders such as MDD, thereby paving the way for more targeted therapeutic strategies.

## 1 Introduction

Major depressive disorder (MDD) is a highly prevalent psychiatric condition characterized by persistent low mood, loss of interest or pleasure, reduced energy, cognitive impairments, and dysregulation of emotional processing (Otte et al., 2016; Marx et al., 2023). Despite its significant socioeconomic burden worldwide, the underlying pathophysiological mechanisms of MDD remain incompletely understood. Accumulating evidence suggests that dysfunction in stress-related neural circuits and neuroendocrine regulation—particularly hyperactivation of the hypothalamic–pituitary–adrenal (HPA) axis and elevated circulating glucocorticoid levels—plays a central role in the pathogenesis of the disorder (Carroll et al., 1976; Bao et al., 2008; Holsen et al., 2013; Zajkowska et al., 2022; Guo et al., 2025).

Among the brain regions implicated in MDD, the amygdala is critically involved in emotional regulation and stress responsiveness. Functional and structural abnormalities in the amygdala have been consistently reported in patients with MDD, including specific alterations in substructures such as the central nucleus of the amygdala (CeA) and the bed nucleus of the stria terminalis (BNST), collectively referred to as the extended amygdala (Weniger et al., 2006; Hamilton et al., 2008; Young et al., 2014; Qiao et al., 2020; Wang et al., 2020; Frank et al., 2021; Roddy et al., 2021; Dhaher et al., 2022; Liu et al., 2024). These subregions are intricately involved in processing sensory and emotional stimuli, integrating experience-dependent emotional memories, and coordinating behavioral and physiological responses to stress (Walker et al., 2003; Phelps and LeDoux, 2005; Roozendaal et al., 2009; Fox et al., 2015; Janak and Tye, 2015; Shackman and Fox, 2016).

Importantly, the amygdala is not a homogenous structure but is composed of multiple subnuclei with distinct cellular compositions and circuit connectivity. These subnuclei form dense intra- and inter-subnuclear networks and function in a cooperative or complementary manner to mediate diverse emotional and stress-related responses (Dong et al., 2001a; Calhoon and Tye, 2015; Fox et al., 2015; Lebow and Chen, 2016; Zhang et al., 2021; Sun et al., 2023). Recent studies have demonstrated that specific neuronal populations within individual amygdala subnuclei exhibit differential activation and plasticity in response to stress or emotional stimuli (Tovote et al., 2015; Ge et al., 2022; Hochgerner et al., 2023). However, the molecular adaptations occurring at the subnuclear level under chronic stress conditions remain poorly characterized, partly due to technical limitations in achieving anatomically precise molecular analysis.

Rodent models of chronic stress provide a powerful platform for investigating the neurobiological basis of depression-like phenotypes. While several models are available, including chronic social defeat stress, unpredictable chronic mild stress, and chronic pain models, chronic corticosterone (CORT) administration offers a highly controllable and reproducible method that recapitulates the endocrine consequences of sustained HPA axis activation (Wang et al., 2017; Planchez et al., 2019). This model reliably induces depression-like behaviors and facilitates the study of glucocorticoid-driven molecular and cellular changes in the brain.

In the present study, we employed a chronic CORT administration model in mice and utilized a fluorescence-guided microdissection system to precisely isolate five amygdala-related subnuclei: the basolateral amygdala (BLA), the lateral and medial divisions of the CeA (CeL and CeM), and the oval and fusiform nuclei of the BNST (BNSTov and BNSTfu). We conducted comparative transcriptomic analyses using RNA sequencing (RNA-seq) to identify subnucleus-specific transcriptional responses to chronic CORT exposure. Notably, these responses exhibited an unexpectedly high degree of diversity across different subnuclei. This study will advance our understanding of the transcriptional changes underlying stress-induced molecular and circuit-level adaptations in the amygdala, and to contribute to the development of novel diagnostic and therapeutic strategies for stress-related psychiatric disorders, including MDD.

## 2 Materials and methods

### 2.1 Mice

All experiments were conducted in accordance with the Nagoya University Regulations on Animal Care and Use in Research and were approved by the Institutional Animal Care and Use Committee, Nagoya University (approval number R23026). Mice were group-housed and kept under 12-h light/dark cycle (lights on at 8:00) with food and water provided *ad libitum* unless otherwise noted. Wild-type C57BL/6J mice were purchased from SLC Japan. *Prkcd-cre* mice {Tg(Prkcd-glc-1/CFP,-cre)EH124Gsat; stock #011559-UCD} and Ai14 mice {B6. Cg-Gt(ROSA)26Sor tm14(CAG-tdTomato)Hze/J; stock #007914} were obtained from the Mutant Mouse Resource & Research Center and the Jackson Laboratory, respectively, and were bred on a C57BL/6J genetic background. Only male mice were used in this study.

### 2.2 CORT administration

**Figure 1:**
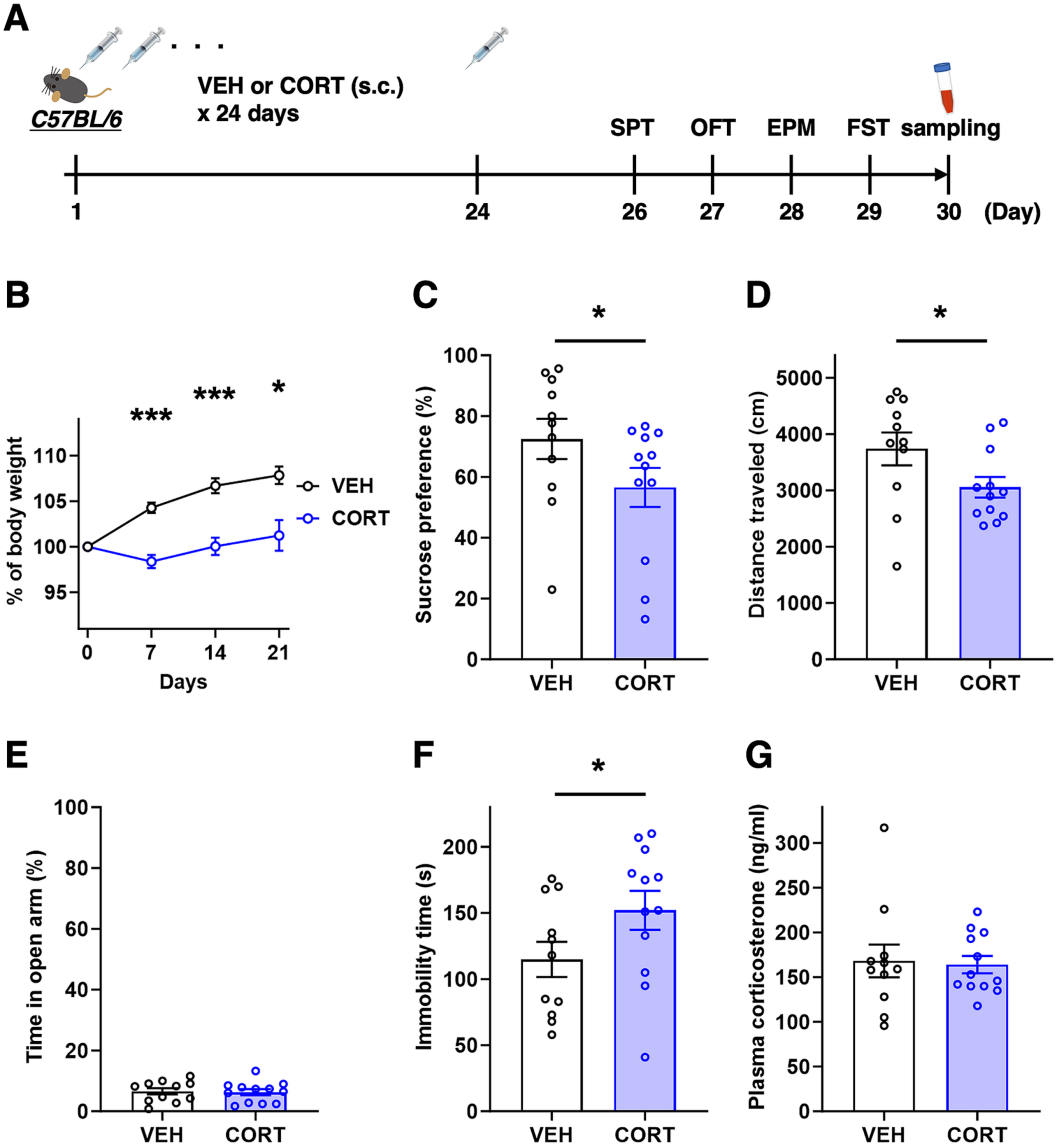
Chronic CORT administered mice exhibit depression-like symptoms. **(A)** Experimental timeline of chronic CORT administration, behavioral testing, and sampling. **(B)** Body weight changes during chronic CORT administration (two-way RM analysis of variance, *F*_interaction_ [3, 63] = 11.87, *p* < 0.001; Sidak’s *post hoc* tests, ***p < 0.001 at day 7 and 14, *p = 0.013 at day 21). **(C)** Percentage of sucrose preference in the SPT (unpaired *t*-test, *p = 0.049). **(D)** Total moving distance during the OFT (unpaired *t*-test, *p = 0.029). **(E)** Percentage of duration in open arm during the EPM test (unpaired *t*-test, *p* = 0.421). **(F)** Total immobility time during the FST (unpaired *t*-test, *p = 0.039). **(G)** Plasma corticosterone level at the experimental day 30 (unpaired *t*-test, *p* = 0.419). VEH, *n* = 11; CORT, *n* = 12.

Corticosterone (27840; Sigma-Aldrich) were suspended in corn oil (8001-30-7; Nacalai Tesque) to a concentration of 20 mg/mL, thoroughly sonicated, and administered subcutaneously at a dose of 40 mg/kg once daily between 10:00 and 12:00, starting at 9–10 weeks of age, according to the schedule shown in Figure 1A. Mice treated with vehicle (VEH, corn oil) on the same schedule were used as controls. Plasma corticosterone levels were measured using the AssayMax Corticosterone ELISA Kit (EC3001-1; AssayPro) according to the instructions provided. Blood samples were collected through the inferior vena cava under deep anesthesia with isoflurane at the time points for measurement, and anticoagulated with sodium citrate. Subsequently, the blood was centrifuged at 3,000 × *g* at 4°C for 10 min, and the supernatant was collected as plasma.

### 2.3 Behavioral tests

#### 2.3.1 Sucrose preference test (SPT)

After the last administration of corticosterone, each mouse was single-housed and acclimated to two bottles with stainless steel sipper tubes (SN-950H; Shinano manufacturing Co.), one containing water and the other containing 1% sucrose solution (on day 24). Following 14 h of water deprivation, each mouse was allowed to drink freely from two bottles, one with water and the other with 1% sucrose solution, from 10:00 to 12:00 (on day 26), and the weight of liquid consumed was measured. Sucrose preference was calculated using the following formula: Sucrose preference (%) = Weight of sucrose solution intake/(Weight of water intake + Weight of sucrose solution intake) × 100.

#### 2.3.2 Open field test (OFT)

The OFT were performed as previously described (Ueda et al., 2021). Briefly, the OF apparatus was a light gray box with dimensions of 40 cm × 40 cm × 30 cm, the spontaneous locomotor activities of the mice were recorded for 10 min, and the total moving distance was calculated using the automatic video tracking software TimeOFCR1 (O’Hara & Co.).

#### 2.3.3 Elevated plus maze test (EPM)

The EPM test were performed as previously described (Ueda et al., 2021). Briefly, EPM consisted of two opposite open arms (25 cm × 5 cm × 0.3 cm) and two enclosed arms (25 cm × 5 cm × 10 cm) elevated to a height of 50 cm above the floor, the activities of the mice were recorded for 10 min, and the time spent on each arm was analyzed using the automatic video tracking software TimeEP1 (O’Hara & Co.).

#### 2.3.4 Forced swim test (FST)

In 2,000 mL glass beakers (150 mm in diameter), approximately 1,500 mL of water at a temperature of 23 to 25°C were filled, and each mouse was allowed to swim for 6 min, which was recorded with a video camera. Before returning to the home cages, mice were dried gently with paper towels and warmed with heating pad for 5 min. The analysis was performed blindly, and the time spent floating without swimming or struggling was measured as immobility time.

### 2.4 Microtissue collection, RNA purification, and cDNA preparation for RNA-seq

Mice were euthanized by cervical dislocation immediately after blood collection, and the brains were isolated and immediately frozen in crushed powder dry ice. Fresh frozen brains were coronally sliced at a 20-μm-thick using Cryostat (CM3050S; Leica), mounted on polyphenylene sulfide Frame Slide (11600294; Leica), and immediately dried up by air blowing. Microtissue dissection was performed using a 110-μm-inner diameter punching needle guided by tdTomato fluorescence under a fluorescence microscope as previously described (Yoda et al., 2017; Ueda et al., 2021). The isolated microtissues were dispensed into the PCR tubes filled with 99.5% ethanol and stored at −80°C until library preparation.

RNA-seq library preparation was performed as previously described (Yamazaki et al., 2020). Briefly, after removing the ethanol using a vacuum evaporator, the isolated microtissues were lysed in 5.3 μL of cell lysis mixture [PKD buffer (QIAGEN): Proteinase K (QIAGEN) = 16:1] at 56°C for 1 h, and the poly(A) RNA was purified with the oligo dT magnetic beads (61,005; Thermo Fisher Scientific) according to the instruction manual. The purified mRNA were directly processed according to the Smart-seq2 protocol (Picelli et al., 2014). PCR products were purified with 0.8 × volume of AMPure XP beads (A63880; Beckman Coulter).

### 2.5 RNA-seq and data analysis

Sequencing and data analysis were conducted as previously described (Yoda et al., 2017). The amplified cDNA (0.25 ng) was used for the preparation of the sequencing library, using the Nextera XT DNA library prep kit (Illumina). The libraries were sequenced with 150-bp paired-end read on an Illumina Hiseq. Adapter sequences were trimmed off from the Raw data (raw reads) of fastq format by flexbar (ver. 3.4.0). The resulting reads were aligned to the Ensembl mouse reference genome (GRCm38 ver. 92) by hisat (ver. 2.1.0) with the default parameters. The number of reads assigned to genes was calculated using featureCounts (ver. 1.6.4). To normalize for the differences in sequencing depth across samples, protein coding gene counts were rescaled to counts per million (CPM) by Trimmed Mean of M-values normalization (TMM) from edgeR. Low-expression genes with an average fewer than 10 CPM in all subnuclei were excluded from analysis. Principal component analysis (PCA) was performed using the prcomp function in R software (ver. 4.5.1) with option scale = TRUE. Differential gene expression analysis was performed using the R/Bioconductor package edgeR (ver. 3.32.0). Differentially expressed genes (DEGs) between VEH and CORT administered mice were defined as *p*-value < 0.01 and |log_2_ FC| > 1.

### 2.6 Protein–protein interaction (PPI) network construction

PPI networks for sets of proteins encoded by all DEGs (including the up and downregulated DEGs) of each subnucleus were constructed using the Cytoscape stringApp (ver. 2.0.2) (Doncheva et al., 2019) with the following settings: network type = full STRING network; confidence score cutoff = 0.4. DEGs (nodes) without any interaction partners within the network are omitted from the visualization. To extract densely interconnected regions in the networks, we used the MCODE plugin (ver. 2.0.3) (Bader and Hogue, 2003) to find clusters with the following settings: include loops = true; degree cutoff = 2; haircut = false; fluff = false; node score cutoff = 0.2, max. Depth = 5, k-core = 2.

### 2.7 Gene ontology (GO) enrichment analysis

The GO enrichment analyses for sets of up or downregulated DEGs of each subnucleus were carried out and functionally grouped GO term networks were constructed using the ClueGO (version 2.5.10)/CluePedia (ver. 1.5.10) plugins (Bindea et al., 2009) in the Cytoscape (ver. 3.10.1) software (Shannon et al., 2003) with the following settings: ontology source = GO_BiologicalProcess-EBI-UniProt-GOA-ACAP-ARAP_25.05.2022, GO_CellularComponent-EBI-UniProt-GOA-ACAP-ARAP_25.05.2022, and GO_Molecularunction-EBI-UniProt-GOA-ACAP-ARAP_25.05.2022; evidence codes = All_Experimental; GO term fusion = true; statistical test used = Enrichment (Right-sided hypergeometric test); *p*-value cutoff = 0.05; GO tree interval level = 4 to 8; minimum number of genes for GO term selection = 3; minimum % of genes for GO term selection = 0; network connectivity Kappa score cutoff = 0.4; GO term grouping = true.

### 2.8 Statistics

All values were expressed as mean ± s.e.m. Two-way RM analysis of variance followed by Sidak’s multiple comparison tests or unpaired one-tailed t-tests were performed using the GraphPad Prism software (ver. 10). One-tailed tests were used for behavioral comparisons because we hypothesized that chronic CORT administration should induce depression-like behavioral changes in a specific, predicted direction. *p*-values smaller than 0.05 were considered statistically significant.

## 3 Results

### 3.1 Chronic CORT administration induces depression-like behaviors in mice

To deepen the understanding of the pathophysiology of MDD, various rodent models have been developed, which recapitulate the psychological and/or physical stress exposure or reflect the physiological changes observed in patients with depression (Wang et al., 2017; Planchez et al., 2019). Among these, chronic CORT administration model mimics the elevated blood glucocorticoid concentration resulting from HPA axis activation and has been reported to display depressive symptoms in both rats and mice (Gregus et al., 2005; Johnson et al., 2006; Zhao et al., 2008). We first confirmed that a single dose of CORT significantly elevated plasma CORT levels under our experimental conditions (Supplementary Figure 1). Subsequently, as shown in Figure 1A, chronic CORT administration was initiated at 9–10 weeks of age and continued daily for 24 days, followed by an evaluation of depression-like symptoms. During the 24-day administration period, mice treated with vehicle (VEH) displayed a gradual increase in body weight, whereas CORT-treated mice exhibited suppressed weight gain (Figure 1B). In behavioral experiments, CORT-treated mice exhibited a decrease in sucrose preference in the SPT, indicative of anhedonia, a reduction in spontaneous locomotor activity in the OFT, suggesting reduced mobility and lethargy, and an increase in immobility time during the FST, indicative of despair-like behavior (Figures 1C,D,F). On the other hand, no significant differences were observed in anxiety-related behavior in the EPM test (Figure 1E). Taken together, these physical and behavioral alterations demonstrate that our chronic CORT-administered mouse model exhibit the face validity as a model of MDD.

### 3.2 Subnucleus-specific transcriptomic changes following chronic CORT exposure

The amygdala is a central brain region that plays a critical role in the emotional processing such as fear and anxiety in response to external stimuli. It is located upstream of the paraventricular nucleus of the hypothalamus and is involved in the activation of the HPA axis, thus playing a significant role in the stress response (Feldman and Weidenfeld, 1998; Herman et al., 2003; Sah et al., 2003; Roozendaal et al., 2009; Janak and Tye, 2015). Glucocorticoids released by the activation of the HPA axis act on both mineralocorticoid and glucocorticoid receptors in the amygdala, providing a concentration-dependent feedback mechanism that modulates amygdala activity (Herman et al., 2005). Consistently, recent studies have reported abnormalities in the activity and connectivity of the amygdala in patients with depression, strongly suggesting a relevance between depression symptoms and dysregulation of amygdala functions (Drevets, 2001; Ressler and Mayberg, 2007; Price and Duman, 2019). However, the amygdala is not a single functional unit. When the central extended amygdala, which encompasses the CeA and the lateral part of the BNST (BNSTL), is included, it consists of numerous subnuclei that are further interconnected through intra- and inter-subnuclear circuits (Fox et al., 2015; Lebow and Chen, 2016). The molecular mechanisms underlying depression within these amygdala subregions remain poorly understood due to this anatomical and functional complexity.

**Figure 2:**
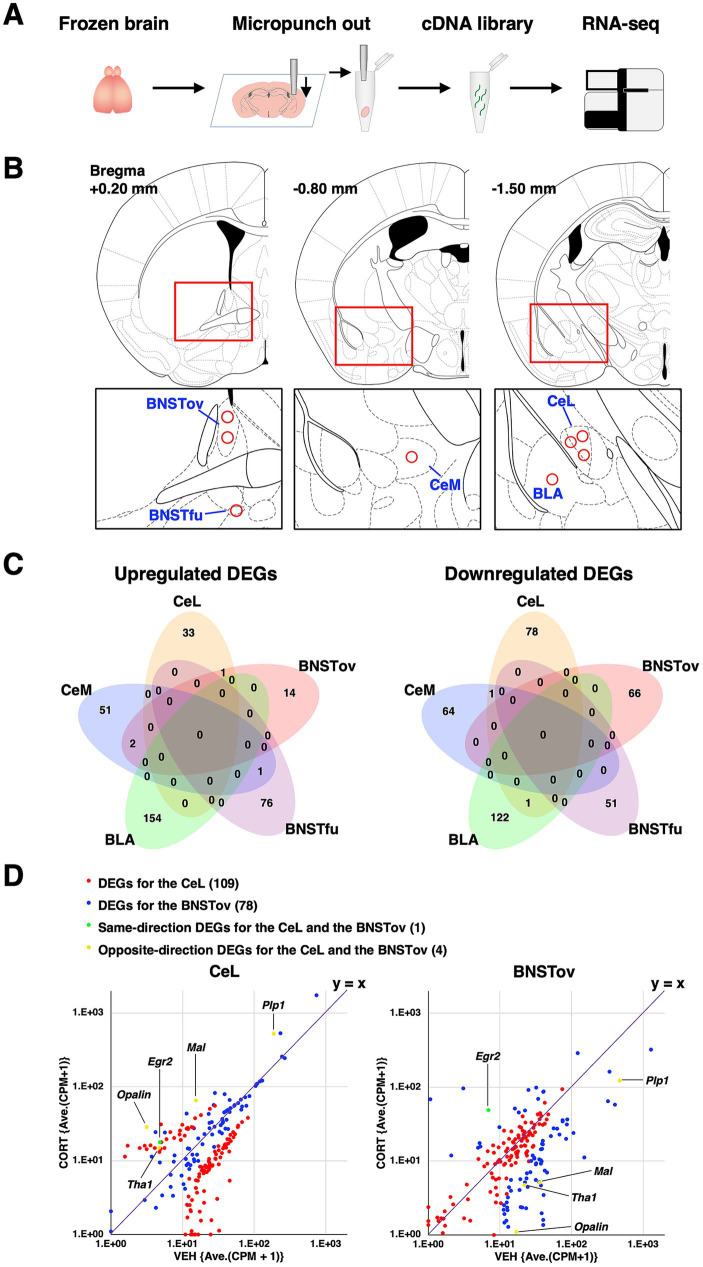
Overview of RNA-seq and DEGs in chronic CORT administered mice. **(A)** Schematic diagram of the experimental workflow, from brain sampling to RNA-seq analysis. **(B)** Schematic brain atlas illustrations showing the eight collection points across five amygdala-related subnuclei obtained via micropunching needle. **(C)** Venn diagram showing the overlap of upregulated and downregulated DEGs across the five amygdala-related subnuclei. **(D)** Scatterplots representing the averaged CPM fold changes between VEH and CORT for DEGs exclusively in the CeL (red), exclusively in the BNSTov (blue), shared in the CeL and the BNSTov in same direction (green), and shared in the CeL and the BNSTov in opposite direction (yellow). Numbers in parentheses indicate the number of DEGs in each category.

To unravel the molecular responses occurring in the amygdala with such complex subnuclear organization, we previously established a microdissection punching system with a punching needle (inner diameter of 110 μm) under a fluorescence microscope, enabling subnucleus-targeted tissue collection and RNA extraction from small tissue samples. We then conducted RNA-seq analysis and successfully captured the transcriptome profiles of each subnucleus (Yoda et al., 2017; Yamazaki et al., 2020; Ueda et al., 2021). Using the same approach, we analyzed gene expression profiles in five amygdala-associated subnuclei—BLA, CeL, CeM, BNSTov, and BNSTfu—following chronic CORT administration, using three biological replicates per group (VEH: *n* = 3, CORT: *n* = 3) (Figures 2A,B, and Supplementary Figure 2A). These subnuclei were selected based on their known involvement in emotional processing and stress-related circuits. For precise subnuclei-targeted tissue collection in this method, we utilized *Prkcd-cre; Ai14* mice, in which PKCδ+ neurons were labeled with tdTomato fluorescence. This allowed us to identify the CeL and BNSTov, where PKCδ+ neurons are abundant, and the CeM and BNSTfu, which receive abundant axonal projections from PKCδ+ neurons (Dong et al., 2001b; Ueda et al., 2021). Similar to wild-type mice, *Prkcd-cre; Ai14* mice also exhibited suppressed body weight gain and depression-like behaviors following chronic CORT administration (Supplementary Figures 2B–D). Since tissue collection was performed 6 days after the final CORT administration, when plasma CORT concentrations had decreased to levels comparable to or lower than those in the VEH group (Figure 1G and Supplementary Figure 2E), it is likely that the direct effects of CORT on gene expression profiles were minimized at the time of sampling. This delayed collection was intentional, as our study aimed to investigate transcriptional changes accompanying established depression-like phenotypes induced by chronic stress exposure, rather than to assess transient pharmacological responses to CORT.

### 3.3 Each amygdala-related subnucleus displayed distinct gene expression responses to chronic CORT administration

Principal component analysis (PCA) of the RNA-seq data revealed that samples from each amygdala-related subnucleus formed separate clusters, indicating both the high precision of our subnucleus-targeted tissue collection and the robustness of the resulting molecular profiling (Supplementary Figure 3A). Moreover, consistent with our previous findings (Ueda et al., 2021), PCA revealed overall molecular similarities in gene expression profiles between CeL and BNSTov, as well as between CeM and BNSTfu. Next, from the results of RNA-seq, we identified differentially expressed genes (DEGs) between VEH and CORT administered mice (defined as *p*-value < 0.01 and |log_2_ FC| > 1) in each of the five subnuclei. As a result, 154 upregulated and 123 downregulated DEGs in the BLA, 34 upregulated and 80 downregulated DEGs in the CeL, 54 upregulated and 65 downregulated DEGs in the CeM, 17 upregulated and 66 downregulated DEGs in the BNSTov, and 77 upregulated and 51 downregulated DEGs in the BNSTfu were extracted, respectively (Figure 2C, Supplementary Figures 3B–F, and Supplementary Table 1). Further examination of overlapping DEGs across subnuclei revealed minimal commonalities. Only four genes were shared among upregulated DEGs: one between the CeL and BNSTov (*Egr2*), two between the CeM and BNSTov (*Arsk* and *Nr4a1*), and one between the CeM and BNSTfu (*Kcnf1*). Among downregulated DEGs only two genes were shared: one between the BLA and CeL (*F3*), and another between the CeL and CeM (*Ttn*) (Figure 2C). These findings suggest that chronic CORT administration induces specific molecular responses in each amygdala subnucleus.

Given our previous report demonstrating the high similarity in the basal gene expression profiles between the CeL and BNSTov, but distinct gene expression responses following the stressful condition of fear conditioning (Ueda et al., 2021), we hypothesized that these regions might exhibit differential transcriptional responses to external stimuli. We visualized the magnitude and direction of gene expression changes induced by chronic CORT administration using scatter plots. Interestingly, most BNSTov-specific DEGs (blue) clustered near the line of symmetry (*y* = *x*) in the CeL plot, indicating that these genes were not differentially expressed in the CeL under CORT treatment. Similarly, most CeL-specific DEGs (red) also showed little variation in the BNSTov plot. Notably, while only one gene (*Egr2*) showed concordant upregulation in both regions (green), we identified four genes (*Plp1*, *Mal*, *Opalin*, and *Tha1*) that exhibited opposite regulation between the CeL and BNSTov (yellow).

Taken together, these findings underscore the complex and region-specific transcriptional adaptations in response to chronic CORT exposure in the amygdala-related subnuclei. In order to gain deeper insights into distinct biological alterations in each subnucleus, we performed protein–protein interaction (PPI) network analysis and gene ontology (GO) enrichment analysis on the identified DEGs in each amygdala-related subnucleus. The detailed results for each region are presented in the following subsections.

### 3.4 BLA: implications of altered excitatory–inhibitory synaptic balance following chronic CORT exposure

**Figure 3:**
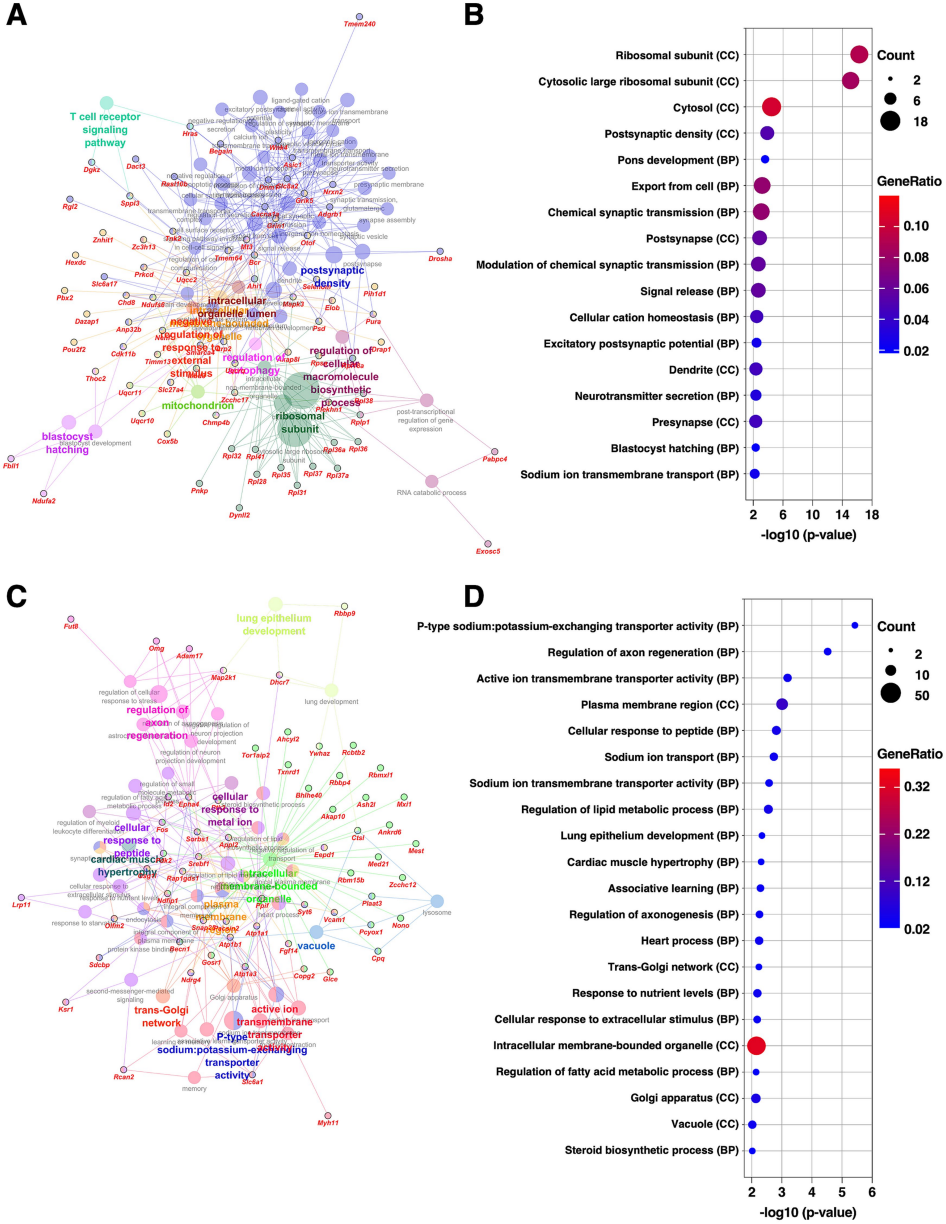
Gene expression changes in the BLA of the mice subjected to chronic CORT administered. **(A,C)** GO enrichment analysis of DEGs extracted from the BLA using the ClueGO plugin. Panels **(A,C)** show enrichment networks for upregulated and downregulated DEGs, respectively. Large, outlineless nodes represent GO terms, where node size corresponds to statistical significance (*p*-value), node color indicates GO term category, and bold labels denote the most significant term within each category (see Supplementary Table 3). Small, black-bordered nodes represent DEGs associated with each GO term, with gene names displayed in red italics. **(B,D)** Dot plots summarizing GO results for **(A,C)**, respectively. Dot size reflects the number of DEGs associated with each term, dot color indicates GeneRatio, and the *x*-axis shows *p*-values. GO terms with *p* < 0.01 are shown in order of significance. The complete dot plots of GO terms with *p* < 0.05 are provided in Supplementary Figures 4B,C.

The BLA is a central hub for integrating affective, sensory, and cognitive information by possessing reciprocal connections with the prefrontal cortex, sensory cortices, and hippocampus. Through these connections, the BLA contributes to the emotional learning and stress regulation (Zhang et al., 2021). It also serves as a major upstream nucleus to the central extended amygdala including the CeA and BNSTL, a key output region of the amygdala involved in emotional expression. Protein–protein interaction (PPI) network analysis was performed on the DEGs extracted in the BLA using the Cytoscape stringApp (Doncheva et al., 2019), and clustering analysis was conducted with the MCODE plugin (Bader and Hogue, 2003). This analysis revealed eight distinct clusters within the network (Supplementary Figure 4A and Supplementary Table 2). The largest cluster (29 DEGs, nodes outlined in green) consisted mainly of ribosomal proteins, all of which were upregulated. The second-largest cluster (13 DEGs, nodes outlined in magenta) comprised genes involved in the oxidative phosphorylation pathway, with most showing increased expression. The third-largest cluster (13 DEGs, nodes outlined in orange) contained genes associated with synaptic function and the Ras/MAPK signaling pathway. Notably, among the synapse-related genes, those involved in inhibitory transmission, such as *Gad1*, *Gad2*, and *Slc6a1*, were downregulated, while excitatory synapse-associated genes, including *Grin1*, *Nrxn2*, and *Cacna1a*, were upregulated, indicating altered excitatory–inhibitory synaptic balance. Subsequently, GO enrichment analysis was conducted separately for the upregulated and downregulated DEGs using the ClueGO plugin in Cytoscape (Bindea et al., 2009). Consistent with the PPI findings, the upregulated DEGs were significantly enriched for GO terms related to ribosomal function and synaptic transmission (Figures 3A,B, Supplementary Figure 4B, and Supplementary Table 3). In contrast, the downregulated DEGs were significantly associated with GO terms linked to ion transporter activity and axonal regulation (Figures 3C,D, Supplementary Figure 4C, and Supplementary Table 3). These findings suggest that chronic CORT administration induces transcriptional changes in the BLA, including upregulation of ribosomal biogenesis, mitochondrial energy production, and synaptic remodeling, potentially shifting the excitatory–inhibitory balance in emotional processing circuits.

### 3.5 CeL: implication of enhanced oligodendrocyte differentiation and myelination following chronic CORT exposure

**Figure 4:**
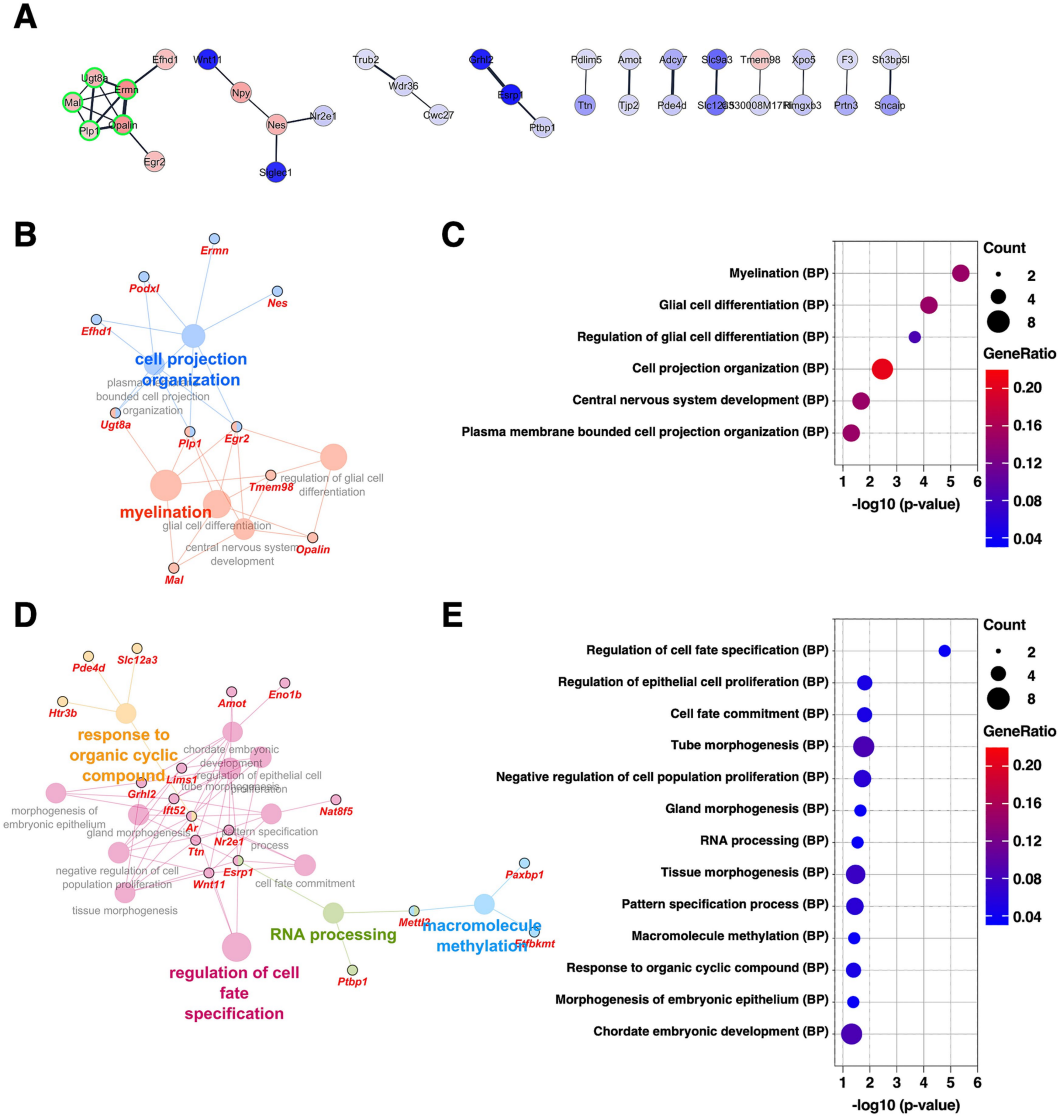
Gene expression changes in the CeL of the mice subjected to chronic CORT administered. **(A)** PPI network of all DEGs extracted from the CeL, visualized using the Cytoscape stringApp. Each node represents a DEG, with red indicating upregulation and blue indicating downregulation; the color intensity reflects the magnitude of log_2_ FC. Edge thickness represents the STRING confidence score for interactions. Nodes outlined in green indicate a cluster identified by the MCODE plugin (see Supplementary Table 2). **(B–E)** GO enrichment analysis of DEGs using the ClueGO plugin. Panels **(B,D)** show enrichment networks for upregulated and downregulated DEGs, respectively. Large, outlineless nodes represent GO terms, where node size corresponds to statistical significance (*p*-value), node color indicates GO term category, and bold labels denote the most significant term within each category (see Supplementary Table 3). Small, black-bordered nodes represent DEGs associated with each GO term, with gene names displayed in red italics. Panels **(C,E)** are dot plots summarizing GO results for **(B,D)**, respectively. Dot size reflects the number of DEGs associated with each term, dot color indicates GeneRatio, and the *x*-axis shows *p*-values. GO terms with *p* < 0.05 are shown in order of significance.

Subsequent analysis was conducted on the DEGs extracted in the CeL. The CeL is a subdivision of the CeA and serves as a major target of input from the BLA as well as from external and internal sensory pathways (Gilpin et al., 2015). It plays a critical role in the expression of emotional and stress-related responses by projecting either directly or via the CeM to brainstem and hypothalamic regions (Moscarello and Penzo, 2022). PPI network analysis revealed a cluster (five DEGs, nodes outlined in green) comprising genes associated with myelination, such as *Opalin*, *Mal*, and *Plp1*, all of which were upregulated (Figure 4A and Supplementary Table 2). Consistent with this, GO analysis of the upregulated DEGs identified terms related to myelination and glial cell differentiation (Figures 4B,C, and Supplementary Table 3). In contrast, GO analysis of the downregulated DEGs revealed terms related to regulation of cell fate specification (Figures 4D,E, and Supplementary Table 3). Notably, *Wnt11*, one of the DEGs annotated with this GO term, is a component of the Wnt/β-catenin signaling pathway, which is known to suppress oligodendrocyte differentiation (Shimizu et al., 2005; Ye et al., 2009). Taken together, these findings suggest that chronic CORT administration promotes oligodendrocyte differentiation and myelin formation in the CeL.

### 3.6 CeM: transcriptional changes suggesting altered peptidergic signaling following chronic CORT exposure

**Figure 5:**
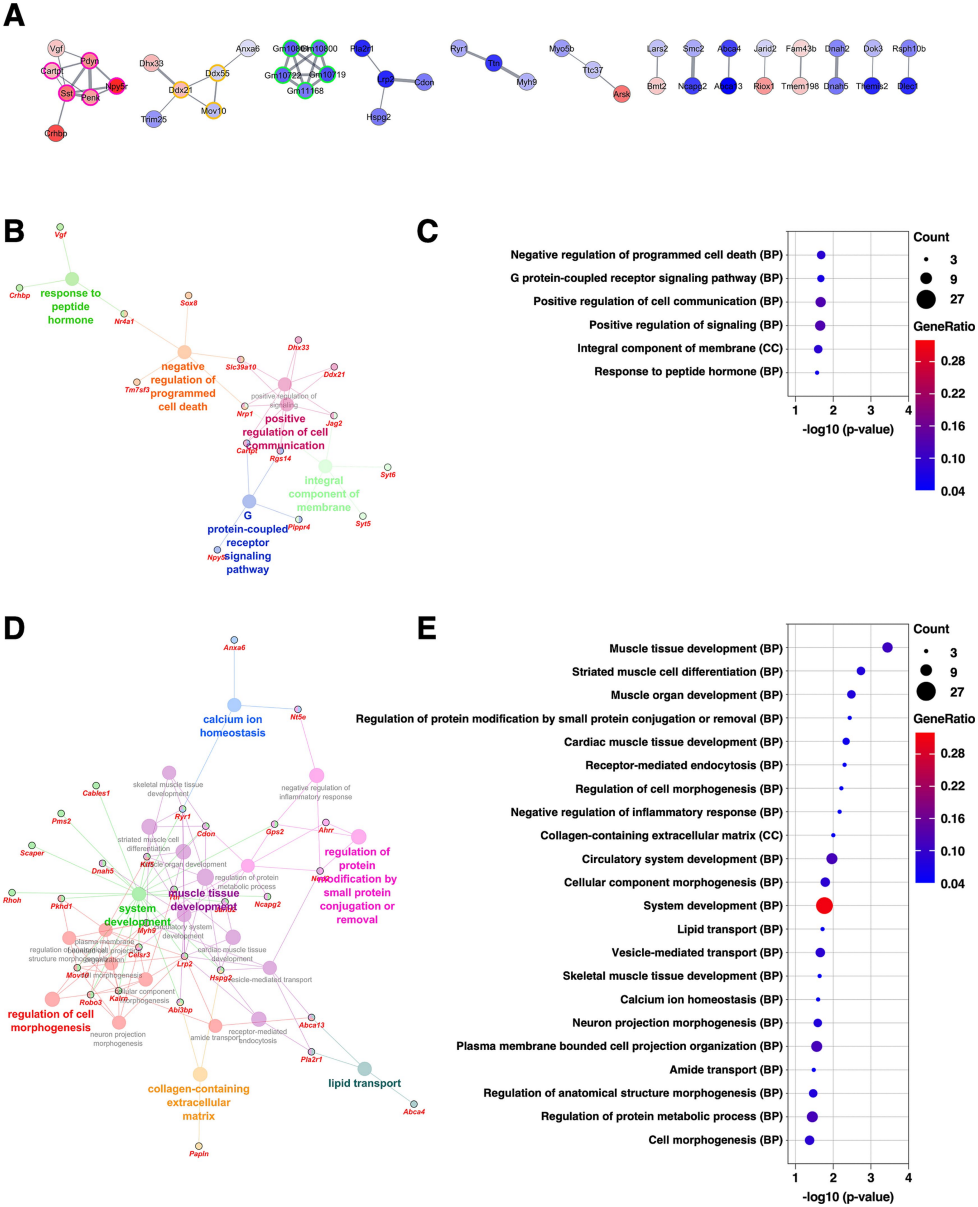
Gene expression changes in the CeM of the mice subjected to chronic CORT administered. **(A)** PPI network of all DEGs extracted from the CeM, visualized using the Cytoscape stringApp. Each node represents a DEG, with red indicating upregulation and blue indicating downregulation; the color intensity reflects the magnitude of log_2_ FC. Edge thickness represents the STRING confidence score for interactions. Nodes outlined in color indicate clusters identified by the MCODE plugin (see Supplementary Table 2). **(B–E)** GO enrichment analysis of DEGs using the ClueGO plugin. Panels **(B,D)** show enrichment networks for upregulated and downregulated DEGs, respectively. Large, outlineless nodes represent GO terms, where node size corresponds to statistical significance (*p*-value), node color indicates GO term category, and bold labels denote the most significant term within each category (see Supplementary Table 3). Small, black-bordered nodes represent DEGs associated with each GO term, with gene names displayed in red italics. Panels **(C,E)** are dot plots summarizing GO results for **(B,D)**, respectively. Dot size reflects the number of DEGs associated with each term, dot color indicates GeneRatio, and the x-axis shows *p*-values. GO terms with *p* < 0.05 are shown in order of significance.

The CeM is another subdivision of the CeA, located medially to the CeL, and is the main output subnucleus of the amygdala, regulating behavioral responses to stress and fear (Gilpin et al., 2015). Among the clusters identified through PPI analysis for DEGs in the CeM, the second-largest cluster (five DEGs, nodes outlined in magenta) consisted of peptide hormones and their receptors, including *Npy5r*, *Sst*, *Pdyn*, *Penk*, and *Cartpt*, all of which were upregulated (Figure 5A and Supplementary Table 2). Consistent with this finding, GO analysis (Figure 5B-E) about the upregulated DEGs revealed terms associated with response to peptide hormone and G protein–coupled receptor signaling pathway (Figures 5B,C, and Supplementary Table 3). Because the CeM contains a diverse population of peptide-producing neurons, which communicate with downstream targets through peptidergic transmission (Wang et al., 2023), these results suggest that chronic stress may enhance peptidergic signaling, potentially modulating CeM output to hypothalamic and brainstem effectors involved in emotional and neuroendocrine control.

### 3.7 BNSTov: attenuation of myelination-related gene expression following chronic CORT exposure

**Figure 6:**
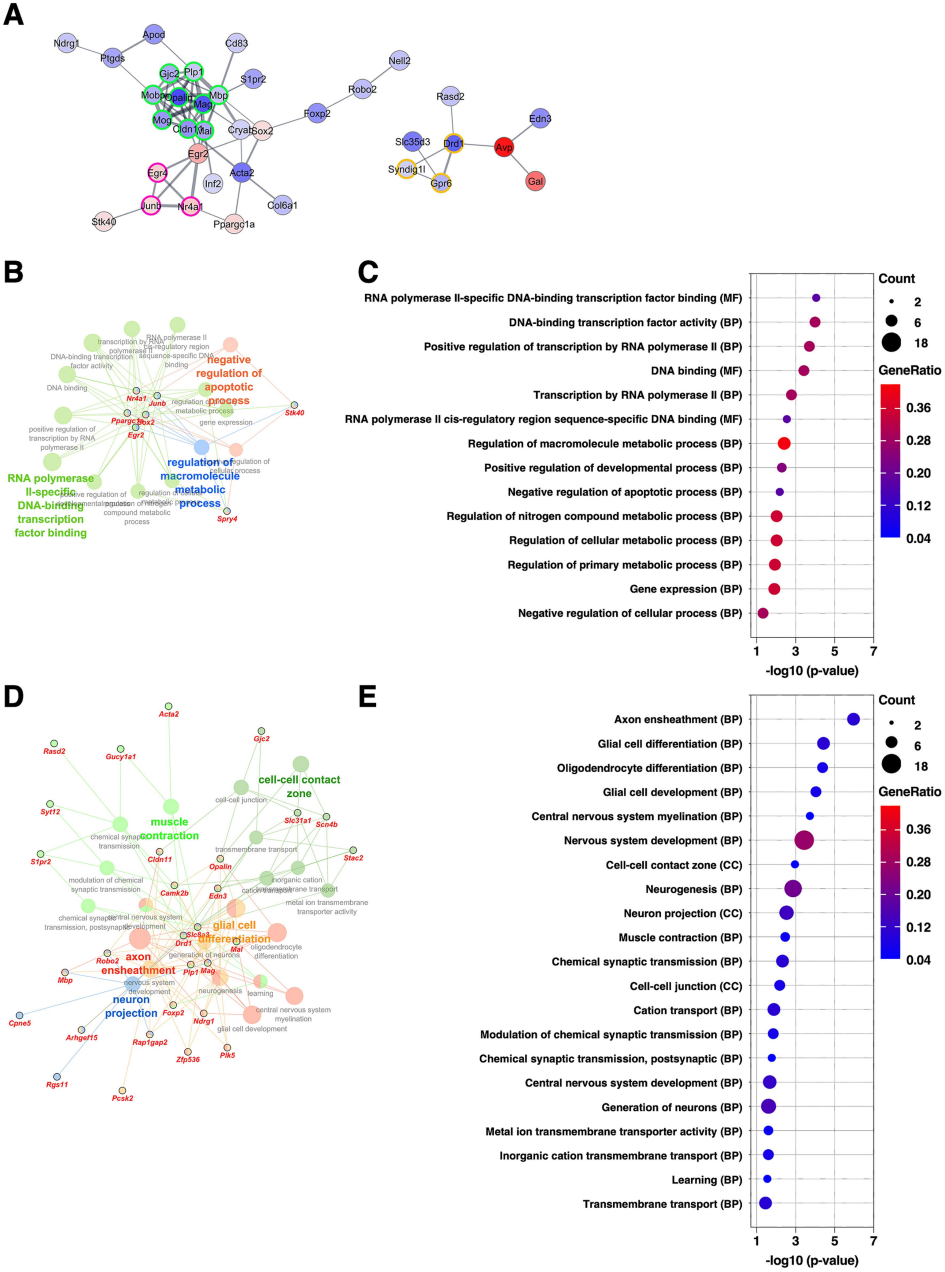
Gene expression changes in the BNSTov of the mice subjected to chronic CORT administered. **(A)** PPI network of all DEGs extracted from the BNSTov, visualized using the Cytoscape stringApp. Each node represents a DEG, with red indicating upregulation and blue indicating downregulation; the color intensity reflects the magnitude of log_2_ FC. Edge thickness represents the STRING confidence score for interactions. Nodes outlined in color indicate clusters identified by the MCODE plugin (see Supplementary Table 2). **(B–E)** GO enrichment analysis of DEGs using the ClueGO plugin. Panels **(B,D)** show enrichment networks for upregulated and downregulated DEGs, respectively. Large, outlineless nodes represent GO terms, where node size corresponds to statistical significance (*p*-value), node color indicates GO term category, and bold labels denote the most significant term within each category (see Supplementary Table 3). Small, black-bordered nodes represent DEGs associated with each GO term, with gene names displayed in red italics. Panels **(C,E)** are dot plots summarizing GO results for **(B,D)**, respectively. Dot size reflects the number of DEGs associated with each term, dot color indicates GeneRatio, and the x-axis shows *p*-values. GO terms with *p* < 0.05 are shown in order of significance.

The BNSTL is anatomically continuous with the CeA, and is considered a core component of the central extended amygdala. Through dense reciprocal neural connections, the BNSTL and CeA are thought to function in a cooperative and complementary manner (Dong et al., 2001b; Fox et al., 2015). The BNSTov is a subdivision of the BNSTL and is known to share similar cellular composition and molecular properties with the CeL (Fox et al., 2015; Ueda et al., 2021). PPI analysis of DEGs extracted from the BNSTov revealed that the largest cluster (nine DEGs, nodes outlined in green) consisted of various well-known myelin-related genes, including *Opalin*, *Mag*, *Cldn11*, *Mog*, *Mal*, *Gjc2*, *Mobp*, *Mbp*, and *Plp1*, all of which were downregulated (Figure 6A and Supplementary Table 2). As shown in Figures 2D, 4A, several of these genes were also identified as upregulated DEGs in the CeL, indicating that these genes are regulated in the opposite direction in the two subnuclei. The second-largest cluster (three DEGs, nodes outlined in magenta) consisted of *Egr4*, *JunB*, and *Nr4a1*, all classified as immediate early genes, and all of which were upregulated. Consistent with these results, GO enrichment analysis of the upregulated DEGs highlighted terms associated with transcriptional regulation, while the downregulated DEGs were enriched for terms related to myelination and oligodendrocyte differentiation (Figures 6B–E and Supplementary Table 3). These findings suggest that the BNSTov and CeL may undergo region-specific and opposing transcriptional regulation in response to chronic stress, particularly in pathways related to oligodendrocyte differentiation and myelination.

### 3.8 BNSTfu: implications of enhanced astrocyte differentiation following chronic CORT exposure

**Figure 7:**
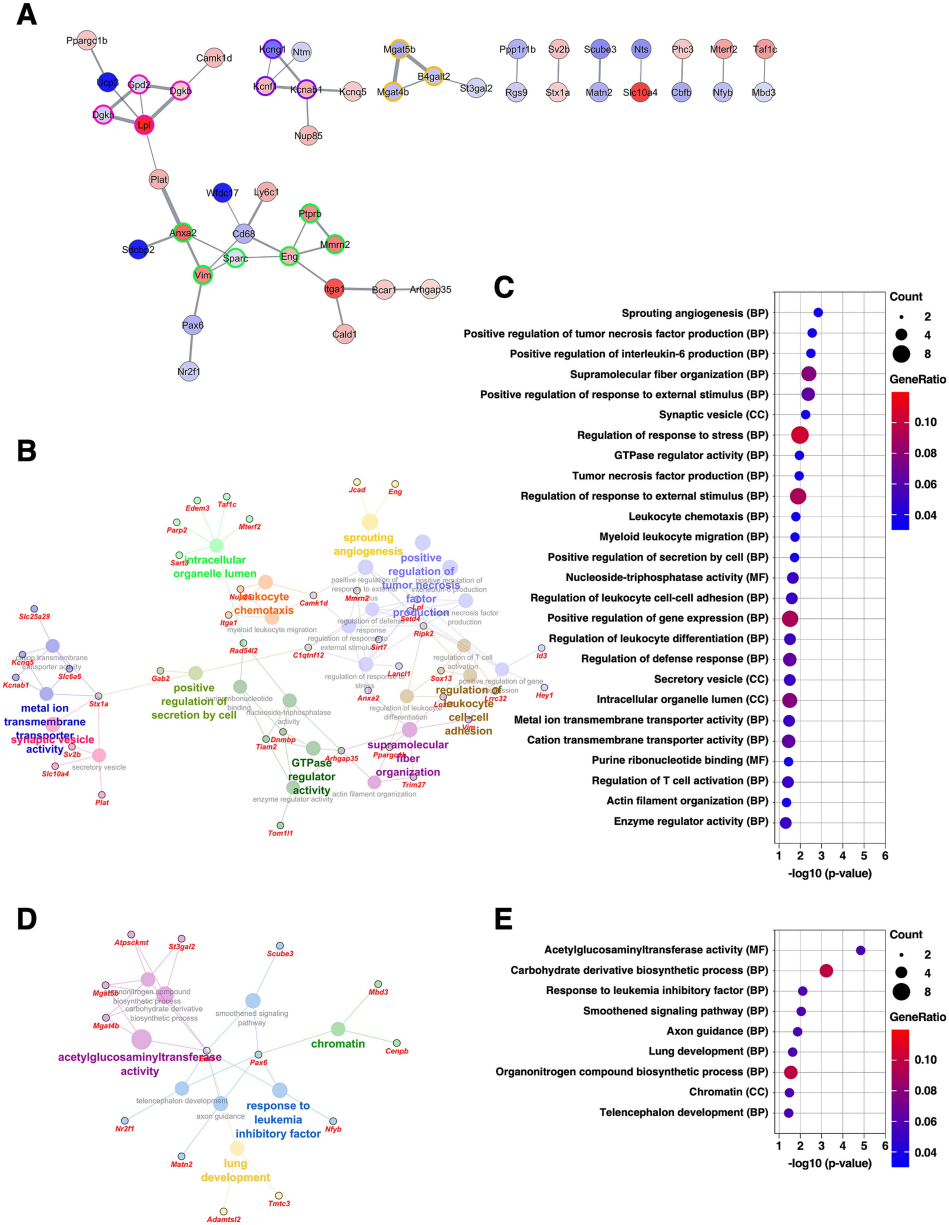
Gene expression changes in the BNSTfu of the mice subjected to chronic CORT administered. **(A)** PPI network of all DEGs extracted from the BNSTfu, visualized using the Cytoscape stringApp. Each node represents a DEG, with red indicating upregulation and blue indicating downregulation; the color intensity reflects the magnitude of log_2_ FC. Edge thickness represents the STRING confidence score for interactions. Nodes outlined in color indicate clusters identified by the MCODE plugin (see Supplementary Table 2). **(B–E)** GO enrichment analysis of DEGs using the ClueGO plugin. Panels **(B,D)** show enrichment networks for upregulated and downregulated DEGs, respectively. Large, outlineless nodes represent GO terms, where node size corresponds to statistical significance (*p*-value), node color indicates GO term category, and bold labels denote the most significant term within each category (see Supplementary Table 3). Small, black-bordered nodes represent DEGs associated with each GO term, with gene names displayed in red italics. Panels **(C,E)** are dot plots summarizing GO results for **(B,D)**, respectively. Dot size reflects the number of DEGs associated with each term, dot color indicates GeneRatio, and the x-axis shows *p*-values. GO terms with *p* < 0.05 are shown in order of significance.

The BNSTfu is also a subdivision of the BNSTL and receives dense projections from the BNSTov as well as from the CeA (Dong et al., 2001b). We have previously reported that the BNSTfu shares molecular properties similar to those of the CeM (Ueda et al., 2021). PPI analysis of DEGs identified in the BNSTfu revealed four clusters. The largest cluster (six DEGs, nodes outlined in green) included *Anxa2* and *Vim*, known markers of reactive astrocytes, as well as *Ptprb*, *Mmrn2*, and *Eng*, which are markers of vascular endothelial cells. The fourth-largest cluster (three DEGs, nodes outlined in purple) consisted of modifier and regulatory subunits of voltage-gated potassium channels, including *Kcng1*, *Kcnf1*, and *Kcnab1* (Figure 7A and Supplementary Table 2). GO analysis of the upregulated DEGs revealed significant enrichment of GO terms related to angiogenesis, interleukin-6 (IL-6) signaling, ion transporters, and synaptic vesicle function (Figures 7B–E and Supplementary Table 3). These findings suggest that, in the BNSTfu, chronic CORT administration promotes differentiation into reactive astrocytes through IL-6–mediated mechanisms, and further imply that changes in vascular structure and synaptic properties may also be occurring.

## 4 Discussion

In this study, we investigated the transcriptional alterations induced by chronic CORT administration across distinct subnuclei of the amygdala. By combining a high-precision 110-μm diameter size microdissection punching system with RNA-seq, we successfully identified subnucleus-specific transcriptional changes. Importantly, we demonstrated that each subnucleus exhibited unique transcriptional alterations in response to chronic CORT exposure, emphasizing the molecular heterogeneity of stress response within the amygdala and the necessity of anatomically resolved analyses to understand the molecular pathophysiology of stress-related disorders.

To model depressive symptoms, we employed the chronic CORT administration model, which mimics sustained activation of the HPA axis observed in patients with MDD (Nestler et al., 2002). While other stress-based models—such as chronic social defeat stress, unpredictable chronic mild stress, and chronic pain—are also widely used, these paradigms often introduce variability due to the difficulty in controlling stress intensity uniformly. Moreover, sensory stimuli inherent to some models (e.g., pain or physical aggression) may directly influence brain activity independent of HPA axis activation, making it difficult to dissociate from stress-induced effect. In contrast, the chronic CORT model enables controlled, reproducible activation of the HPA axis without sensory pathway interference. Notably, CORT-treated mice in this study did not exhibit anxiety-like behaviors, suggesting that behavioral phenotypes vary with the trait, intensity, and duration of stress exposure. Further studies applying similar gene expression analyses to other depression models will be essential to clarify how molecular changes relate to behavioral phenotypes across different stress contexts.

We chose a microdissection punching method for transcriptomic profiling due to its ability to isolate specific amygdala subnuclei with high anatomical precision. Our previous work demonstrated that this approach reliably captures molecular signatures at the subnucleus level (Yoda et al., 2017; Yamazaki et al., 2020; Ueda et al., 2021). In contrast, gene expression analyses at the whole-brain or brain-region level may drop subnucleus-specific changes due to signal dilution. While single-cell transcriptomics has made great advances recently, comprehensive gene expression profiling with preserved spatial information remains technically challenging, especially for genes with low expression levels. Thus, combining our high-resolution dissection-based techniques with emerging single-cell or spatial transcriptomic approaches may offer complementary insights across multiple scales.

In the BLA, we observed gene expression changes indicative of a shift in the excitatory–inhibitory (E/I) balance, characterized by downregulation of inhibitory synapse-associated genes and upregulation of excitatory ones. This aligns with previous reports showing BLA hyperactivity in both animal models of depression and human patient studies (Drevets, 2003; Becker et al., 2023; Asim et al., 2024a). Notably, alterations in specific inhibitory neuronal populations within the BLA have been implicated in the emergence of depression- and anxiety-like behaviors (Asim et al., 2024b). These findings support the notion that the transcriptional alterations we observed—particularly those indicating reduced inhibitory synaptic components—may contribute to circuit-level E/I imbalance, leading to behavioral changes. Moreover, increased expression of ribosomal protein genes may reflect enhanced protein synthesis necessary for dendritic and synaptic remodeling, which has been observed in the BLA under chronic stress. Genes involved in oxidative phosphorylation were also upregulated, potentially representing an adaptive response to heightened metabolic demands accompanying elevated neural activity and protein synthesis (Hall et al., 2012; Rumpf et al., 2023). Although these findings suggest functional changes such as altered synaptic plasticity and increased metabolic activity, it is important to note that these interpretations are predictions based on transcriptomic data. Future studies incorporating functional analyses, such as electrophysiological recordings, will be necessary to validate whether these transcriptional changes are indeed associated with functional alterations. Additionally, recent studies have revealed that BLA neurons exhibit considerable genetic and spatial heterogeneity, with their different subpopulations playing distinct roles (O'Leary et al., 2020; Lim et al., 2024). This spatially heterogenous organization suggests that discrete neuronal ensembles within the BLA may contribute to specific aspects of stress-related behavior and responses. Our subnucleus-level transcriptomic approach, while anatomically broader than cell-type–specific profiling, provides complementary information by capturing the aggregate molecular adaptations within each anatomically defined subregion. Integrating such fine-scale cellular heterogeneity with subnucleus-level gene expression changes in future studies could yield a more comprehensive understanding of BLA functional organization in stress-related disorders.

Interestingly, we identified distinct patterns of glial cell responses across subnuclei. We found that myelin-related gene expression changed in opposite directions in the CeL and BNSTov. In the CeL, chronic CORT administration led to upregulation of genes associated with oligodendrocyte differentiation and myelination. This finding is consistent with our previous reports of gene expression changes in the CeL following fear conditioning (Ueda et al., 2021), suggesting that stress-related stimuli can promote myelination in this subnucleus. In contrast, the BNSTov, which shares similar constitutive molecular profiles with the CeL at the basal level (Ueda et al., 2021), exhibited downregulation of the same myelin-related genes. These opposing patterns suggest that these subnuclei are differentially engaged in the response to chronic stress, and that myelin regulation may be circuit-selective, potentially modulating specific input/output pathways in a regionally targeted manner. Again, these interpretations are predictive based on molecular signatures, and functional consequences should be confirmed experimentally. Supporting evidence from previous literature, however, suggests that activity-dependent myelination is a plausible downstream effect of the observed gene expression changes (Lee and Fields, 2009; Bonetto et al., 2021). Future investigations are needed to determine which circuits are selectively myelinated or demyelinated in response to chronic stress.

Furthermore, in the BNSTfu, we found increased expression of genes associated with reactive astrocytes and vascular remodeling. Reactive astrocytes are known to contribute to neuroprotection, synaptic remodeling, and inflammatory responses (Chung et al., 2015; Becerra-Calixto and Cardona-Gómez, 2017; Ding et al., 2021; Lawrence et al., 2023; Zhao et al., 2024). Together with the observed changes in oligodendrocyte/myelin-related genes in the CeL and BNSTov, our data suggest that diverse glial cell responses occur in a subnucleus-specific manner across the central extended amygdala. Such glial plasticity may contribute to circuit-level adaptations under chronic stress and could represent novel targets for diagnostic and therapeutic interventions.

Several limitations of this study should be noted. Although we achieved high-precision spatial transcriptome profiling at the subnucleus scale across anatomically defined regions, we did not directly assess corresponding functional or morphological changes, such as synaptic activity, circuit connectivity, or glial types and morphology. Validation of transcriptomic findings using RT-qPCR, *in situ* hybridization, or protein expression analysis is important but was not performed in this study. Follow-up research to validate expression changes of key DEGs and to assess their functional consequences remains to be conducted in future studies. Additionally, sex differences were not explored in this study but are known to influence both HPA axis function and stress susceptibility. Expanding this work to include female animals and to various stress models will be essential for a more comprehensive understanding of stress-related brain pathophysiology. Furthermore, although minimal overlap was observed in DEGs across subnuclei, chronic CORT exposure may affect coordinated transcriptional networks that are not detectable by differential expression analysis alone. While this study focused on subregion-specific DEGs, future investigations employing coexpression-based approaches could provide additional insight into shared or interacting molecular programs across the amygdala subregions.

In conclusion, our study highlights the importance of anatomically resolved molecular analyses in uncovering the subnucleus-specific responses to chronic stress. By integrating region-specific transcriptomics with behavioral phenotyping, we advance toward identifying the molecular substrates underlying stress-induced behavioral alterations with refined resolution, which may inform future strategies for the diagnosis and treatment of affective disorders.

## Data Availability

The RNA-seq datasets presented in this study can be found in online repositories. The names of the repository/repositories and accession number(s) can be found: https://www.ddbj.nig.ac.jp/, DRR703261–DRR703308. Other datasets generated and/or analyzed during the current study are available from the corresponding author at reasonable request.

## Supplementary material

SUPPLEMENTARY TABLE 1 List of DEGs.

SUPPLEMENTARY TABLE 2 List of MCODE clusters.

SUPPLEMENTARY TABLE 3 Results of GO analysis.
