# Supplementary material for: Divergent and subnucleus-specific gene expression responses to chronic stress hormone exposure in the amygdala
Source: Front Mol Neurosci. 2025 Sep 12;18:1659846. doi: 10.3389/fnmol.2025.1659846 (PMC12463952; doi:10.3389/fnmol.2025.1659846)
Supplement: Supplementary file 4 [file Data_Sheet_1.PDF]

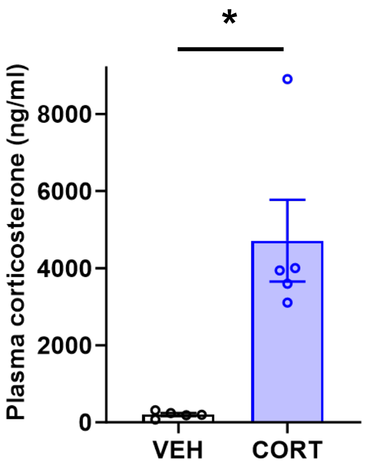

Supplementary Figure 1. Acute effect on plasma corticosterone levels following a single CORT injection. Plasma corticosterone concentrations measured one hour after a single subcutaneous injection of corticosterone (unpaired t-test, ** p = 0.0014). VEH, n = 5; CORT, n = 5.

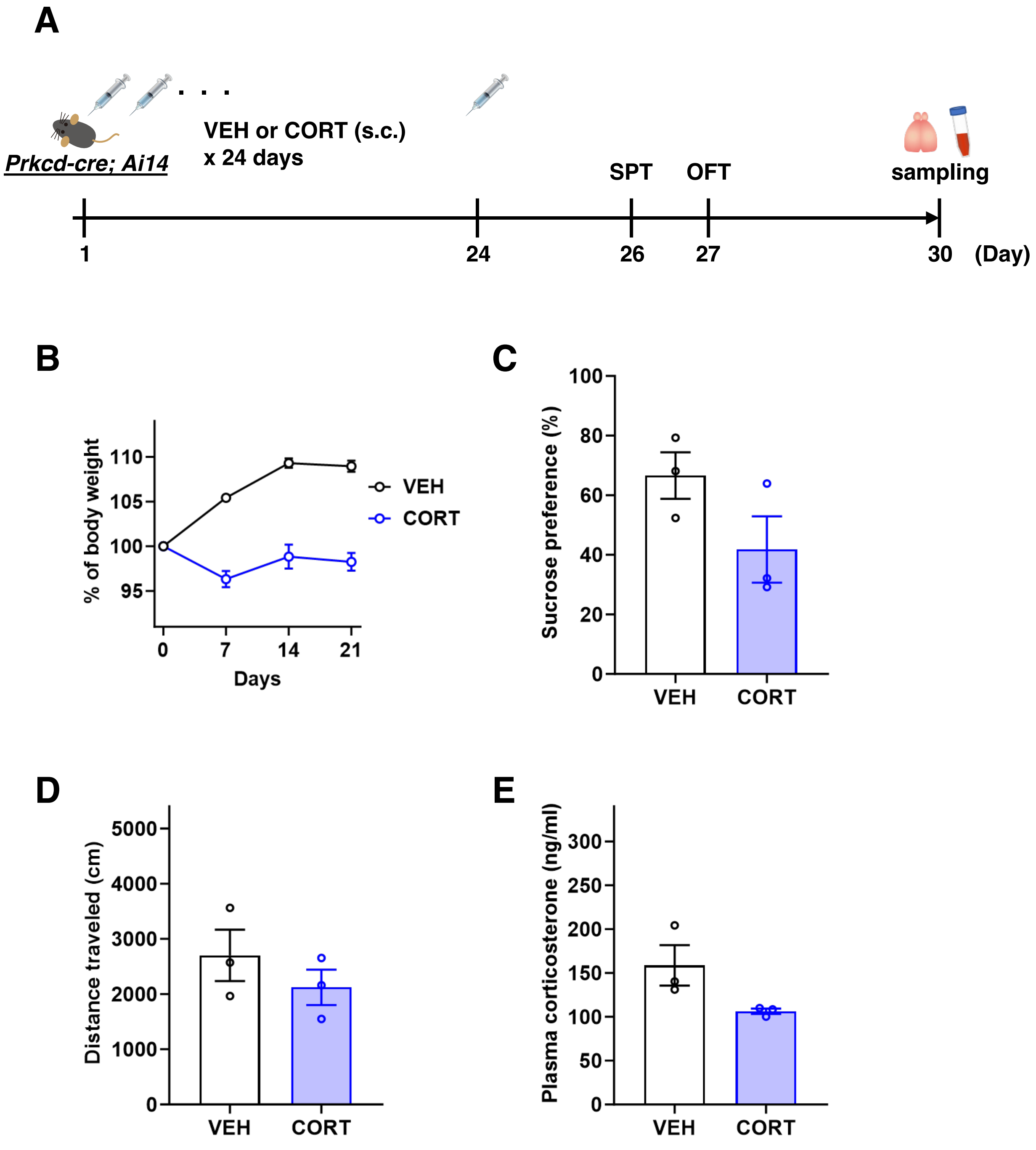

Supplementary Figure 2. Effects of chronic CORT administration on *Prkcd-cre; Ai14* mice used for RNA-seq analysis. (A) Experimental timeline of chronic CORT administration, behavioral testing, and sampling. (B) Body weight changes during chronic CORT administration. (C) Percentage of sucrose preference in the SPT. (D) Total moving distance during the OFT. (E) Plasma corticosterone level at the experimental day 30. VEH, n = 3; CORT, n = 3.

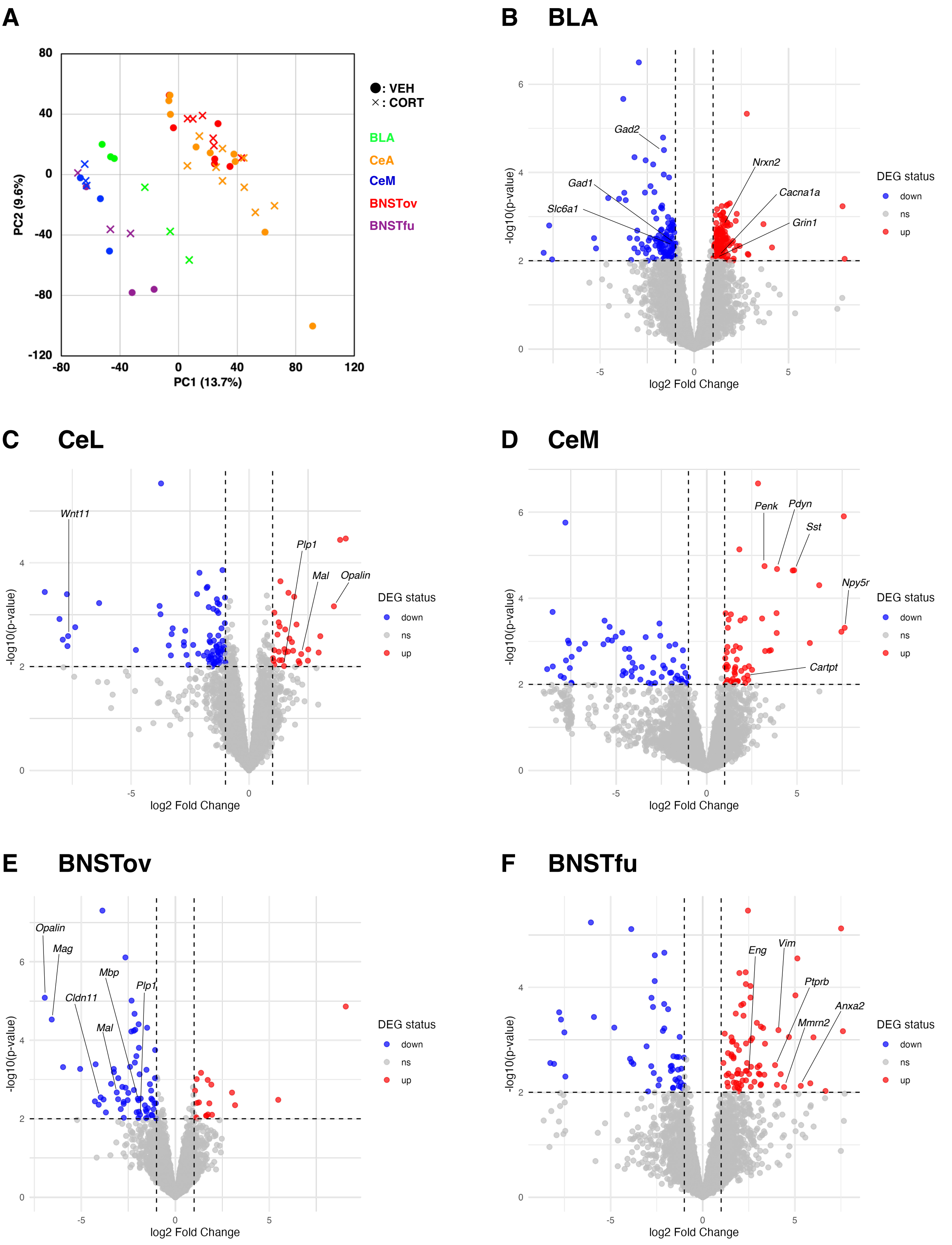

Supplementary Figure 3. Gene expression profiling of amygdala-related subnuclei from chronic CORT administered mice. (A) Principal component analysis (PCA) of RNA-seq data illustrating inter-sample variance and clustering of gene expression profiles across amygdala-related subnuclei. (B-F) Volcano plots showing DEGs between VEH and CORT administered mice in each subnucleus. Red dots indicate upregulated DEGs, and blue dots indicate downregulated DEGs.

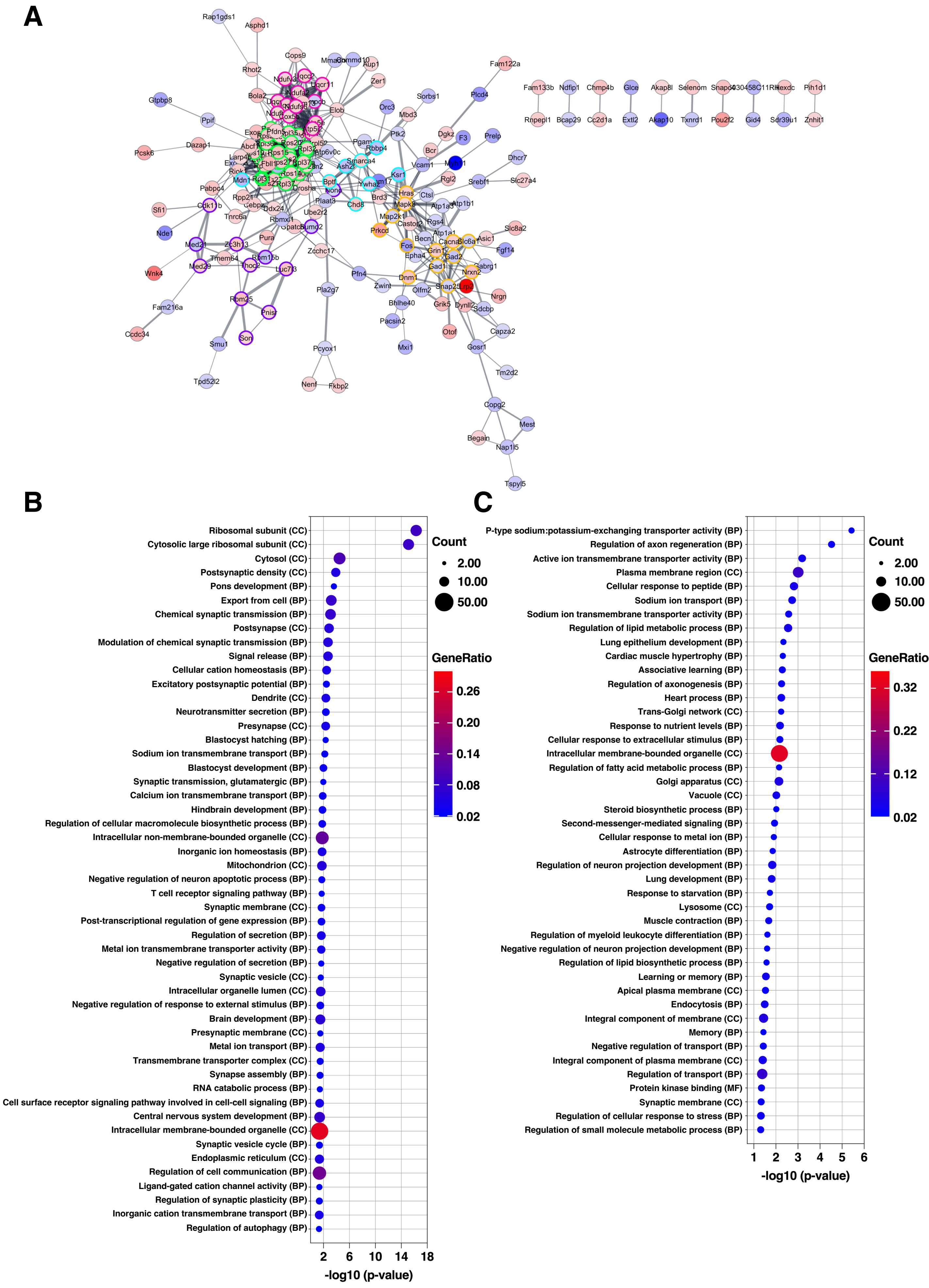
A
Rap1gds1
Asphd1
Cops9
Rhot2
Gtpbp8
Pcsk6
Dazap1
Pabpc4
Sfi1
Nde1
Wnk4
Fam216a
Ccdc34
Smu1
Tpd52l2
Cdk11b
Med21
Med29
Zc3h13
Tmem64
Thoc2
Rbm15b
Luc7l3
Rbm25
Pnisl
Son
Tnrc6a
Pura
Zcchc17
Pla2g7
Pcyox1
Nenf
Fkbp2
Pfn4
Zwint
Bhlhe40
Pacsin2
Mxi1
Olfm2
Otof
Grik5
Dynll2
Sdcbp
Capza2
Gosr1
Tm2d2
Copg2
Mest
Begain
Nap1l5
Tspyl5
Fam122a
Plcd4
Sorbs1
Orc3
Mbd3
Zer1
Aup1
Elob
Ptk2
Dgkz
F3
Prelp
Dhcr7
Slc27a4
Srebf1
Bcr
Vcam1
Rgl2
Ksr1
Hras
Ctsl
Mapk8
Atp1a3
Atp1b1
Rgs4
Castor2
Prkcd
Fos
Becn1
Atp1a1
Slc6a1
Asic1
Slc8a2
Fgf14
Grin1
Gad2
Gad1
Gabrg1
Nrxn2
Dnm1
Snap25
Nrgn
Epha4
Chd8
Brd3
Smarca4
Ash2l
Rbbp4
Pgam
Atp6v0c
Plaat3
Ube2r2
Sumo2
Drosha
Nono
Bptf
Mdn1
Rpp21
Cebpz
Ddx24
Rbmxl1
Gpatch
Fam133b
Rnpepl1
Ndfip1
Bcap29
Chmp4b
Cc2d1a
Glce
Extl2
Akap8l
Akap10
Selenom
Txnrd1
Pou2f2
Gid4
Hexdc
Sdr39u1
Pih1d1
Znhit1
B
Ribosomal subunit (CC)
Cytosolic large ribosomal subunit (CC)
Cytosol (CC)
Postsynaptic density (CC)
Pons development (BP)
Export from cell (BP)
Chemical synaptic transmission (BP)
Postsynapse (CC)
Modulation of chemical synaptic transmission (BP)
Signal release (BP)
Cellular cation homeostasis (BP)
Excitatory postsynaptic potential (BP)
Dendrite (CC)
Neurotransmitter secretion (BP)
Presynapse (CC)
Blastocyst hatching (BP)
Sodium ion transmembrane transport (BP)
Blastocyst development (BP)
Synaptic transmission, glutamatergic (BP)
Calcium ion transmembrane transport (BP)
Hindbrain development (BP)
Regulation of cellular macromolecule biosynthetic process (BP)
Intracellular non-membrane-bounded organelle (CC)
Inorganic ion homeostasis (BP)
Mitochondrion (CC)
Negative regulation of neuron apoptotic process (BP)
T cell receptor signaling pathway (BP)
Synaptic membrane (CC)
Post-transcriptional regulation of gene expression (BP)
Regulation of secretion (BP)
Metal ion transmembrane transporter activity (BP)
Negative regulation of secretion (BP)
Synaptic vesicle (CC)
Intracellular organelle lumen (CC)
Negative regulation of response to external stimulus (BP)
Brain development (BP)
Presynaptic membrane (CC)
Metal ion transport (BP)
Transmembrane transporter complex (CC)
Synapse assembly (BP)
RNA catabolic process (BP)
Cell surface receptor signaling pathway involved in cell-cell signaling (BP)
Central nervous system development (BP)
Intracellular membrane-bounded organelle (CC)
Synaptic vesicle cycle (BP)
Endoplasmic reticulum (CC)
Regulation of cell communication (BP)
Ligand-gated cation channel activity (BP)
Regulation of synaptic plasticity (BP)
Inorganic cation transmembrane transport (BP)
Regulation of autophagy (BP)
2 6 10 14 18
-log10 (p-value)
Count
2.00
10.00
50.00
GeneRatio
0.26
0.20
0.14
0.08
0.02
C
P-type sodium:potassium-exchanging transporter activity (BP)
Regulation of axon regeneration (BP)
Active ion transmembrane transporter activity (BP)
Plasma membrane region (CC)
Cellular response to peptide (BP)
Sodium ion transport (BP)
Sodium ion transmembrane transporter activity (BP)
Regulation of lipid metabolic process (BP)
Lung epithelium development (BP)
Cardiac muscle hypertrophy (BP)
Associative learning (BP)
Regulation of axonogenesis (BP)
Heart process (BP)
Trans-Golgi network (CC)
Response to nutrient levels (BP)
Cellular response to extracellular stimulus (BP)
Intracellular membrane-bounded organelle (CC)
Regulation of fatty acid metabolic process (BP)
Golgi apparatus (CC)
Vacuole (CC)
Steroid biosynthetic process (BP)
Second-messenger-mediated signaling (BP)
Cellular response to metal ion (BP)
Astrocyte differentiation (BP)
Regulation of neuron projection development (BP)
Lung development (BP)
Response to starvation (BP)
Lysosome (CC)
Muscle contraction (BP)
Regulation of myeloid leukocyte differentiation (BP)
Negative regulation of neuron projection development (BP)
Regulation of lipid biosynthetic process (BP)
Learning or memory (BP)
Apical plasma membrane (CC)
Endocytosis (BP)
Integral component of membrane (CC)
Memory (BP)
Negative regulation of transport (BP)
Integral component of plasma membrane (CC)
Regulation of transport (BP)
Protein kinase binding (MF)
Synaptic membrane (CC)
Regulation of cellular response to stress (BP)
Regulation of small molecule metabolic process (BP)
1 2 3 4 5 6
-log10 (p-value)
Count
2.00
10.00
50.00
GeneRatio
0.32
0.22
0.12
0.02

Supplementary Figure 4. Gene expression changes in the BLA of the mice subjected to chronic CORT administered (related to Figure 3). (A) PPI network of all DEGs extracted from the BLA, visualized using the Cytoscape stringApp. Each node represents a DEG, with red indicating upregulation and blue indicating downregulation; the color intensity reflects the magnitude of log2 FC. Edge thickness represents the STRING confidence score for interactions. Nodes outlined in color indicate top five clusters identified by the MCODE plugin (see Supplementary Table 2). (B, C) Dot plots summarizing GO results for Figures 3A and 3C, respectively. Dot size reflects the number of DEGs associated with each term, dot color indicates GeneRatio, and the x-axis shows *p*-values. GO terms with $p < 0.05$ are shown in order of significance.
